# Clustered Regularly Interspaced Short Palindromic Repeats-Associated Proteins13a combined with magnetic beads, chemiluminescence and reverse transcription-recombinase aided amplification for detection of avian influenza a (H7N9) virus

**DOI:** 10.3389/fbioe.2022.1094028

**Published:** 2023-01-05

**Authors:** Hongpan Xu, Lijun Peng, Jie Wu, Adeel Khan, Yifan Sun, Han Shen, Zhiyang Li

**Affiliations:** ^1^ Nanjing Drum Tower Hospital Clinical College of Jiangsu University, Nanjing, China; ^2^ Clinical Laboratory Center, Affiliated Hangzhou Chest Hospital, Zhejiang University School of Medicine, Hangzhou, Zhejiang, China; ^3^ State Key Laboratory of Bioelectronics, School of Biological Science and Medical Engineering, National Demonstration Center for Experimental Biomedical Engineering Education (Southeast University), Southeast University, Nanjing, China

**Keywords:** H7N9, magnetic beads, LwCas13a, reverse transcription-recombinase aided amplification, chemiluminescence

## Abstract

Clustered Regularly Interspaced Short Palindromic Repeats (CRISPR) and Clustered Regularly Interspaced Short Palindromic Repeats-Associated Proteins (CRISPR-Cas) have promising prospects in the field of nucleic acid molecular diagnostics. However, Clustered Regularly Interspaced Short Palindromic Repeats-based fluorescence detection technology is mainly hindered by proteins with conjugated double bonds and autofluorescence, resulting in high fluorescence background, low sensitivity and incompatible reaction systems, which are not conducive to automatic clinical testing. Chemiluminescence (CL) detection technology has been applied mainly owing to its greatly high sensitivity, as well as low background and rapid response. Therefore, we developed a rapid, ultrasensitive and economical detection system based on Clustered Regularly Interspaced Short Palindromic Repeats-Clustered Regularly Interspaced Short Palindromic Repeats-Associated Proteins 13a combined with magnetic beads (MBs) and chemiluminescence (CL) (Cas13a-MB-CL) to detect Influenza A (H7N9), an acute respiratory tract infectious disease. The carboxyl functionalized magnetic beads (MBs-COOH) were covalently coupled with aminated RNA probe while the other end of the RNA probe was modified with biotin. Alkaline phosphatase labeled streptavidin (SA-ALP) binds with biotin to form magnetic beads composites. In presence of target RNA, the collateral cleavage activity of Cas13a was activated to degrade the RNA probes on MBs and released Alkaline phosphatase from the composites. The composites were then magnetically separated followed by addition of ALP substrate Disodium 2-chloro-5-{4-methoxyspiro [1,2-dioxetane-3,2′-(5′-chloro) tricyclo (3.3.1.13,7) decan]-4-yl}-1-phenyl phosphate (CDP-star), to generate the chemiluminescence signal. The activity of Associated Proteins 13a and presence of target RNA was quantified by measuring the chemiluminescence intensity. The proposed method accomplished the detection of H7N9 within 30 min at 25°C. When combined with Reverse Transcription- Recombinase Aides Amplification (RT-RAA), the low detection limit limit of detection was as low as 19.7 fM (3S/N). Our proposed MB-Associated Proteins 13a-chemiluminescence was further evaluated to test H7N9 clinical samples, showing superior sensitivity and specificity.

## 1 Introduction

Clustered Regularly Interspaced Short Palindromic Repeats (CRISPR) and CRISPR-Associated Proteins (CRISPR-Cas)-based diagnostics have revolutionized in the field of nucleic acid molecular diagnostics (Abudayyeh et al., 2016; Gootenberg et al., 2017; Chen et al., 2018; Khan et al., 2019; Srivastava et al., 2020). Different types of promising CRISPR-Cas based system can combine fluorescence to achieve signal amplification (Abudayyeh et al., 2017; Liang et al., 2019; Peng et al., 2020). However, proteins have conjugated double bonds along with spontaneous fluorescence, thus having a high background (Chang et al., 2019; Guindani et al., 2019). And incompatible reaction systems often need to combine microfluidic or manually open the lid to take samples from the first step reaction, which is not conducive to clinical large-scale sample screening. (Peng et al., 2020; Wang et al., 2020; Zhou et al., 2022).

Chemiluminescence (CL) detection technology has completely avoided the shortcomings of high fluorescent signal background and easy to quench, which is becoming the most important area of biological analysis research and development (Yang et al., 2014; Huang; Zhang et al., 2021; Hu et al., 2022; Liu et al., 2022; Zhao et al., 2022). Also, the CL signal of antibody joint with typical horseradish peroxidase (HRP), Alkaline phosphatase (ALP) and magnetic beads (MBs) platforms have been perfectly employed to commercialized immunoassay diagnosis kits and effectively solving clinical problems (Huang; Speletas et al., 2020; Huang et al., 2019). Besides, methods for using CRISPR-Cas13a combined with CL and MBs have not been reported yet, so it is necessary to use CL with CRISPR-Cas13a to improve sensitivity also low the background.

Avian influenza A (H7N9) virus is classified into subtypes based on two surface proteins, namely Hemagglutinin (HA) and Neuraminidase (NA). It is a new recombinant virus which has the ability to bind to human receptors, and can invade the epithelial cells of human lower respiratory tract and lung cells, and in mammals (Zhou et al., 2013). Since human H7N9 infection was first discovered in 2013, there have been five human infections in China (Chen et al., 2013; Gao et al., 2013). Influenza caused by H7N9 virus has the characteristics of short incubation period and high mortality, which seriously threatens the safety of human life (Gao et al., 2013). Early diagnosis is very important to control the spread of diseases, improve the prognosis of patients and reduce medical expenses. The detection of H7N9 virus mainly includes three methods: 1) virus isolation and culture; 2) Detection of relevant antigens in serum (such as the detection of antigens by ELISA) (Yu et al., 2017); 3) the detection of viral nucleic acid. In recent years, H7N9 virus has been mainly detected by nucleic acid in clinical practice. The conventional nucleic acid detection method is quantitative reverse polymerase chain reaction (RT-qPCR) (Yang et al., 2018). However, it is difficult for grass-roots hospitals to meet the requirements due to the need for specific testing equipment, long time consuming, need of trained operators and special planning of the laboratory. Therefore, it is necessary to establish a reliable, sensitive and rapid H7N9 virus detection system.

**SCHEME 1:**
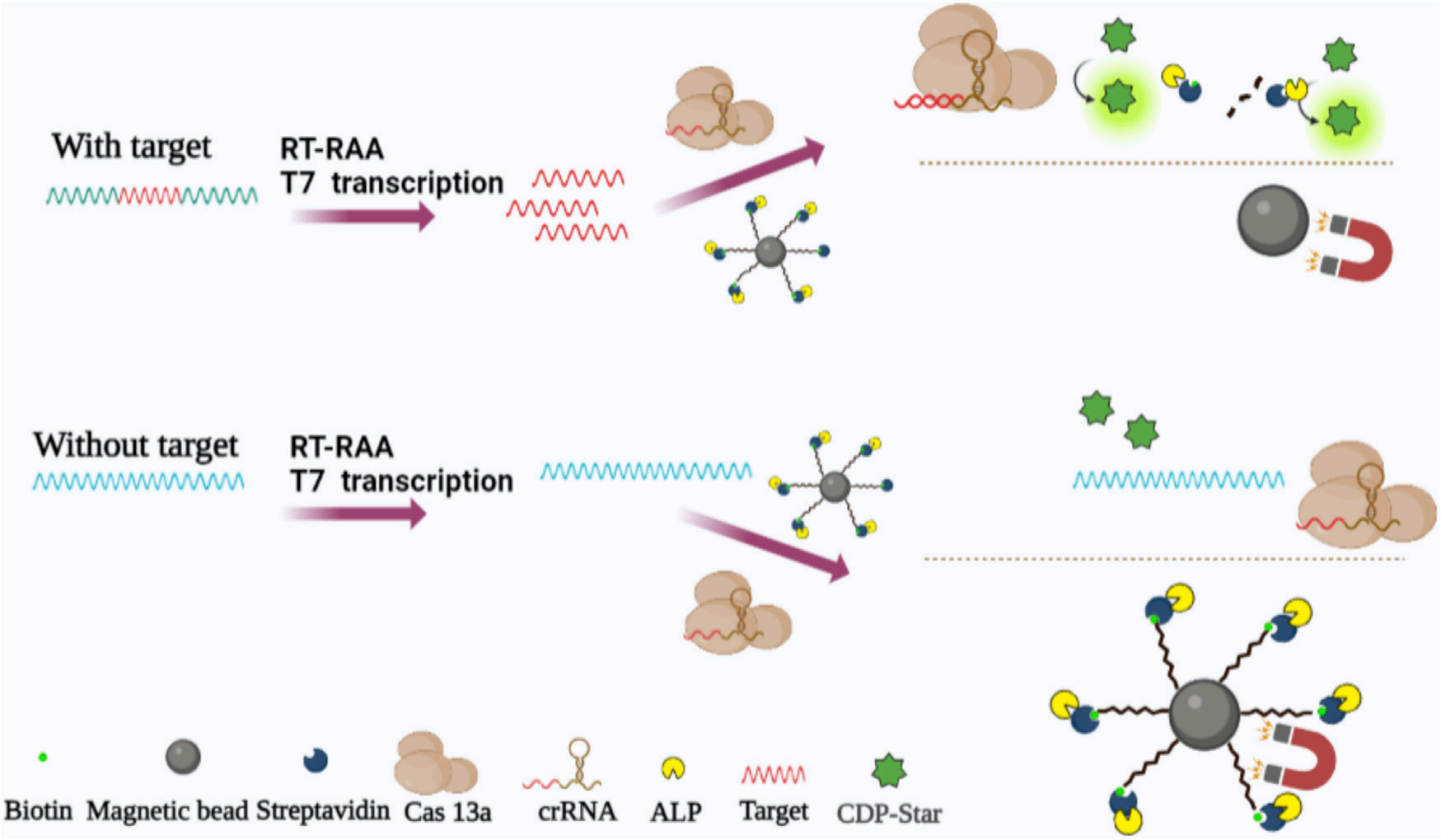
Schematic illustration of RT-TAA-Cas13a-MB-CL platform for the detection of nucleic acids. Target RNA was amplified with RT-RAA. The aminated RNA probes were coupled to MBs-COOH and through biotin conjugation SA-ALP. In addition, Cas13a bound target RNA under the guidance of crRNA degrading RNA probes and releasing ALP. CL signals were generated when added CDP-Star.

In this study, we established a method for detecting H7N9 by recording the chemiluminescence signals of ALP utilizing RT-RAA-Cas13a-MB-CL to amplify nucleic acids. The schematic process can be seen in Scheme 1, a 5′-end amino-modified and 3′-end biotin-modified RNA probe was labeled on a carboxyl functionalized MB (MBs-COOH) by a condensation chemical reaction, and coupled with alkaline phosphatase labeled streptavidin (ALP-SA) to form MB-RNA-ALP complexes. Only when the target RNAs are present, CRISPR RNA (crRNA) of Cas13a binds and activates the collateral cleavage activity of Cas13a to cleave MB-RNA-ALP complexes and release ALP. Then, the solution was separated *via* external magnetic fields, followed by addition of ALP substrate in the supernatant to generate a chemiluminescence signals. When combined with Reverse Transcription- Recombinase Aided Amplification (RT-RAA) to amplify target RNA, this method showed superior sensitivity and specificity in short times.

## 2 Materials

2-morpholinoethanesulfonic acid monohydrate (MES), 1-ethyl-3-(3-dimethylaminopropyl) carbodiimide hydrochloride (EDC·HCl), sodium chloride (NaCl), Tween-20, magnesium chloride (MgCl_2_), peptone, yeast powder, agarose powder, ampicillin (Amp) and ALP-SA were purchased from Dalian Meilun Biological Technology Co., Ltd. (Suzhou, China, http://www.meilune.com). Tris-base, Tris hydrochloric acid (Tris HCl), Isopropyl β-d-1-thiogalactopyranoside (IPTG) and 4-hydroxyethyl piperazine ethanesulfonic acid (HEPES) were supplied by Sigma-Aldrich (St. Louis, United States, https://www.sigmaaldrich.com). Dynabeads^®^ MyOne™ Carboxylic Acid (Life Technologies, United States) and HiScribe T7 Quick High Yield RNA Synthesis kit (New England Biolabs) were also used. RT-RAA nucleic acid amplification kit was purchased from Jiangsu Qitian Gene Biotechnology Co., Ltd. (Wuxi, China, http://www.qt-bio.com). The RNA probes and H7N9 gene were synthesized by was purchased from GenScript Biotech Co., Ltd. (Nanjing, China, https://www.genscript.com.cn/quick_order_menu.html). All the oligonucleotides (Sup. Table 1) and diethylpyrocarbonate (DEPC) treated water were obtained from Sangon (Shanghai, China, https://www.sangon.com). The pC013-Twinstrep-SUMO-huLwCas13a (Addgene plasmid #90097) was provided by Zhang Feng’s laboratory. CDP-Star was served by Sysmex Corporation (Japan, https://www.sysmex.com.cn). All chemical reagents used in the study were of analytical grade and all solutions were prepared with DEPC treated water. All the hemagglutinin (HA) and neuraminidase (NA) sequences of H7N9 used in the present study were downloaded from the NCBI (National Center for Biotechnology Information), and the sequences of RNA probe, crRNA and H7N9 virus and other control viruses, including seasonal influenza viruses (A/H1N1 and A/H3N2), 2009 swine-origin influenza virus A/H1N1, and avian influenza viruses (A/H5N1 and A/H9N1) were donated by Jiangsu Provincial Center for Disease Prevention and Control are shown in Sup. Table 1. And nuclease assay buffer (NAB) was prepared using 20 mM HEPES, 60 mM NaCl, 6 mM MgCl_2_, pH 6.8.

### 2.1 LwCas13a protein expression and purification

Extraction of PCO13-Twinstrep-SUMO-huLwCas13a-plasmid used the Plasmid Mini Kit (OMEGA). 50ng of plasmids were then transferred into E. coli competent cells BL21 (Cwbiotech). After transformation, 100 μL of dilutions of cells were plated on ampicillin (Amp) and Luria-Bertani (LB)-agar plate and incubated overnight at 37°C. The next day, a single colony was picked and plated in a 10 ml of LB medium (Containing 100 μL of Amp), which was used to inoculate 1 L of LB medium and shook overnight at speed of 220 r/min until the OD600 was 0.6 at 37°C. At this time, LwCas13a protein expression was induced by supplementation with IPTG to a final concentration of 500 μM. The culture was maintained at 16°C for 16 h, and the cells were then centrifuged at 4,500 g for 10 min at 4°C. Considering the stability of the Cas13a protein, all steps of protein purification were carried out at 4°C. After centrifugation, the cell pellet was harvested and resuspended in lysis buffer (20 mM Tris-HCl, 500 mM NaCl, 1 mM DTT, pH 7.4). Then, protease inhibitor (MCE) and lysozyme were added, followed by sonication (JX-650, Shanghai, China) with following conditions: 200 W of power, ultrasonic 2.5 s and 10 s of pause for 30 min. Centrifugation was carried at 10,000 g for 60 min at 4°C and filtered the supernatant through a 220 nm filter (Sterile Millex). The filtered supernatant was applied to a 5 ml HisTrapHP nickel ion affinity column (GE Healthcare Life Sciences) *via* Fast protein liquid chromatography (FPLC) (AKTA PURE, GE Healthcare Life Sciences). Then the protein bound column was washed three times using a buffer (20 mM Tris-HCl, 500 mM NaCl, 30 mM imidazole, pH 7.4). About 250 Units of SUMO protease (ThermoFisher) was then loaded onto the column and incubated overnight at 4°C with rotation. Finally, the column was washed with lysis buffer again and the collected solution was analyzed by 12% SDS-PAGE. Fractions containing pure LwCas13a were pooled and exchanged the buffer *via* a Millipore centrifugal ultrafiltration tube (Amicon Ultra) to a storage buffer (600 mM NaCl, 50 mM Tris-HCl, pH 7.5, 5% glycerol, 2 mM DTT). The final product was stored at −80°C.

### 2.2 Preparation of RNA standard and crRNA

Plasmid containing the HA or NA gene of the H7N9 virus was amplified using PrimeSTAR Max DNA Polymerase (TaKaRa), and then purified using a SanPrep PCR product purification kit (Sangon Biotech). Purified dsDNA was incubated with T7 polymerase overnight at 37°C using HiScribe T7 Quick High Yield RNA Synthesis Kit (New England Biolabs). The RNA was purified by RNA rapid concentration and purification kit (Sangon Biotech) and quantified by One drop (American Thermal Power Corporation) to prepare a gradient RNA standard solution. The crRNA was designed based on the target RNA sequence. Two complementary crRNA-DNA primers (with an appended T7 promoter sequence) were annealed with final concentration 10 μM. Then the dsDNAs were transcribed to crRNA, using the HiScribe T7 Quick High Yield RNA Synthesis kit (New England Biolabs).

### 2.3 Preparation of MB-RNA-ALP complexes

Taking 10 mg/ml carboxylated MBs was stored in a refrigerator at 4°C, followed by vortex on a vortex shaker for 5 min to mix the MBs. 100 μL of the mixed MBs were then washed twice with 1 ml of 100 mM MES buffer, resuspended in 800 μL of MES buffer. Then an appropriate amount of 1.25 M EDC and RNA probe were added to make a total volume of 1 ml, rotating and incubated overnight at 4°C. The next day, after blocking the MBs twice with 1 ml of TT buffer (0.25 M Tris, 0.01% Tween -20, pH 8.0, 30 min per round of incubation), the MBs were resuspend with 1 ml of NAB. At last, ALP was added (according to the volume ratio of ALP:MBs = 1:10) to the MBs and rotating for 1 h. The MB-RNA-ALP complexes were washed twice with 1 ml of TT buffer and stored in TT buffer at 4°C.

### 2.4 Chemiluminescent detection of MBs composites to verification of LwCas13a activity

For each test, 2 μL of primary MBs were added to the reaction solution (11.25 nM purified LwCas13a, 22.5 nM crRNA, 10 nM target RNA, 1 μL of murine RNase inhibitor (New England Biolabs), 100 ng of background total human RNA (purified from human PBMC), supplemented with NAB to 50 μL) and mixed on a roller mixer for 30 min. The activated LwCas13a cleaved the RNA probe and the ALP was released from the MB-RNA-ALP complexes. Non-target RNA or non-Cas13a protein served as a negative control (NC) in the detection system, and MBs complexes supplement 50 μL of NAB buffer served as background value. At the same time, MB-RNA-ALP complexes were incubated with RNase acting as a positive control, to confirm that the ALP could release from complexes and act as a signal molecule. After magnetic separation, 30 μL of the reaction solution and ALP substrate were incubated in a 96-well microplate format and the CL signal value was recorded using a microplate reader (SpectraMax M5) at 42°C for 15 min.

### 2.5 HA or NA gene RNA detection with Cas13a-MB-CL system

About 1 μL of mouse RNase inhibitor, 11.25 nM purified LwCas13a, 22.5 nM crRNA, 100 ng of background total human RNA and various concentrations of target RNA were mixed with MBs complexes and were further supplemented with NAB buffer to a final volume of 50 μL for HA RNA and NA RNA detection.

### 2.6 H7N9 detection with RT-TAA-Cas13a-MB -CL system

Improved detection assays were performed with RT-RAA nucleic acid amplification kit (Qitian Gene Biotechnology) and HiScribe T7 Quick High Yield RNA Synthesis kit (New England Biolabs) as follows: 0.48 μM forward primer, 0.48 μM reverse primer, 14 mM magnesium acetate anhydrous, 12.5 μL mM rNTP, 2.5 μLT7 Mix, various concentrations of target RNA, 2 μL murine RNase inhibitor and 25 μL Basic E-mix to 50 μL final volume. After completing optimization of the RT-RAA amplification system for the cleaving system, MB-RNA-ALP complexes were added into the reaction solution (11.25 nM purified LwCas13a, 22.5 nM crRNA, 1 μL RNase inhibitor, 100 ng RNA purified from human PBMC and various concentration gradients of the RT-RAA amplification products of HA RNA) and replenished with NAB buffer 50 μL to a final volume of 50 μL for HA RNA and NA RNA detection.

### 2.7 Specificity and clinical sample testing

H7N9 virus samples and other viruses samples, including seasonal influenza viruses (A/H1N1 and A/H3N2), 2009 swine-origin influenza virus A/H1N1, and avian influenza viruses (A/H5N1 and A/H9N1) donated by Jiangsu Provincial Center for Disease Prevention and Control were tested after amplification by RT-RAA. The testing was conducted using the same procedure as described in Section 2.6.

## 3 Results and discussion

### 3.1 LwCas13a protein expression and purification

**FIGURE 1:**
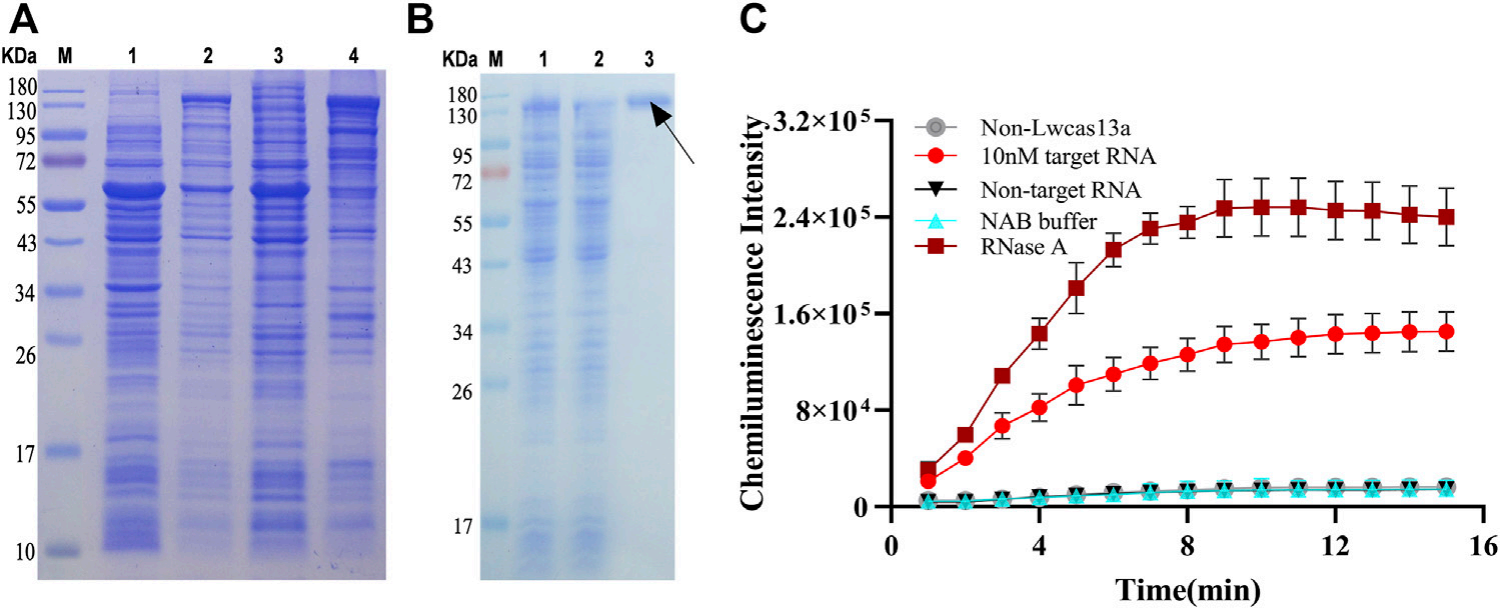
LwCas13a protein expression, purification and verification. **(A)** The expression of LwCas13a protein. M, marker; lane 1, E. coli wasn’t induced by IPTG, lane 2, IPTG induced E. coli overnight; lane 3, precipitation of broken cells; lane 4, supernatant of broken cells. **(B)** The purification of LwCas13a protein. M, marker; lane 1, supernatant of broken cells; lane 2, flow-through treated with SUMO enzyme. Lane 3, Eluent. **(C)** Verification of LwCas13a activity.

In our study, based on the structural features of PCO13-Twinstrep-SUMO-huLwCas13a-plasmid, the LwCas13a protein was purified by the SUMO enzyme. The molecular weight of LwCas13a containing the SUMO site and the marker protein was 155.2 kD, but it was 138.5 kD after digestion with SUMO enzyme. LwCas13a expressed in E. coli was analyzed by SDS-PAGE. Coomassie blue stained gel results showed that the IPTG significantly induced the production of Cas13a protein in E. coli (Figure 1A, lane 2). The bacterial solution after IPTG induction was collected, centrifuged, and the cell pellet was disrupted by sonication and centrifuged again. SDS-PAGE analysis was performed on disrupted cell supernatant and cell pellet. It was found that the LwCas13a was mainly present in the supernatant (Figure 1A, lane three and 4). Because the LwCas13a protein contains a His-tag, we used nickel ion affinity chromatography to adsorb water-soluble proteins. A protein without multiple consecutive histidine was eluted from the column by the wash buffer, while LwCas13a was still on the column, and then the SUMO site in LwCas13a was digested with SUMO enzyme, and the LwCas13a lost 6 × His was easily eluted from the column by lysis buffer, pointed by the black arrow (Figure 1B, lane 3). By this purification method a highly pure LwCas13a protein can be obtained.

### 3.2 Chemiluminescent detection of MBs composites to verification of LwCas13a activity

RNase A can degrade RNA probes, so we used RNase A as a positive control to verify the RNase activity of LwCas13a. As shown in Figure 1C, the CL intensity increased with time and became stable after 10 min approximately. The CL intensity of positive sample (10 nM) was evidently higher than that of negative control (no target HA RNA or LwCas13a) and the blank, which indicated that Cas13a could non-specifically cleave RNA probes by binding the target RNA and its corresponding crRNA.

### 3.3 Optimization of experimental parameters

**FIGURE 2:**
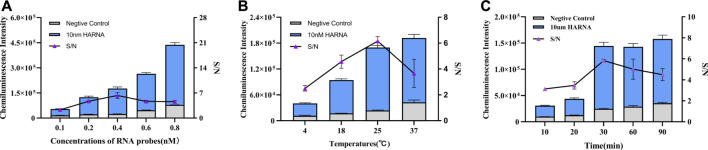
Optimization of experimental conditions. **(A)** Optimization of RNA probe concentration. **(B)** Optimization of reaction temperature. **(C)** Optimization of reaction time. The data are presented as mean ± S.D. of three replicate measurements.

Given the non-specific absorption and steric hindrance effect, the density of probes on the surface of MBs may also affect the cleavage of RNA probes by Cas13a protein. About 1 mg MB and five different concentrations of RNA probes (0.1, 0.2, 0.4, 0.6 and 0.8 nM) were used to determine their effect on the CL intensity. We used 10 nM target RNA to activate Cas13a. Figure 2A showed that the CL intensity increased as the probe concentration increased. However, considering the positive judgment criterion, according to Figure 2A, there was a maximum S/N value at 0.4 nM, so 0.4 nM could be proposed as optimal concentration of RNA probes for the modification of MBs. The reaction temperature was investigated as a factor that may affect the cleaving efficiency of Cas13a. A temperature gradient (4, 18, 25, 37°C) was set to test the effect of the reaction temperature on the CL intensity. Figure 2B illustrated that as the temperature increased, the CL intensity increased, but the negative control and blank also increased. According to the change of S/N value, 25°C was selected as the optimal reaction temperature. Reaction time is another factor that affects the cleaving efficiency of Cas13a. A hybridization time gradient (10, 20, 30, 60, 90 min) was established to examine its effect on CL intensity. Figure 2C showed that in the range of 10–30 min, the CL intensity significantly increased with increased hybridization time and the CL intensity gradually stabilized after 30 min, but the negative control also increased with time. Therefore, 30 min was considered to be the best reaction time.

### 3.4 Sensitivity detection of H7N9 after RT-RAA amplification

**FIGURE 3:**
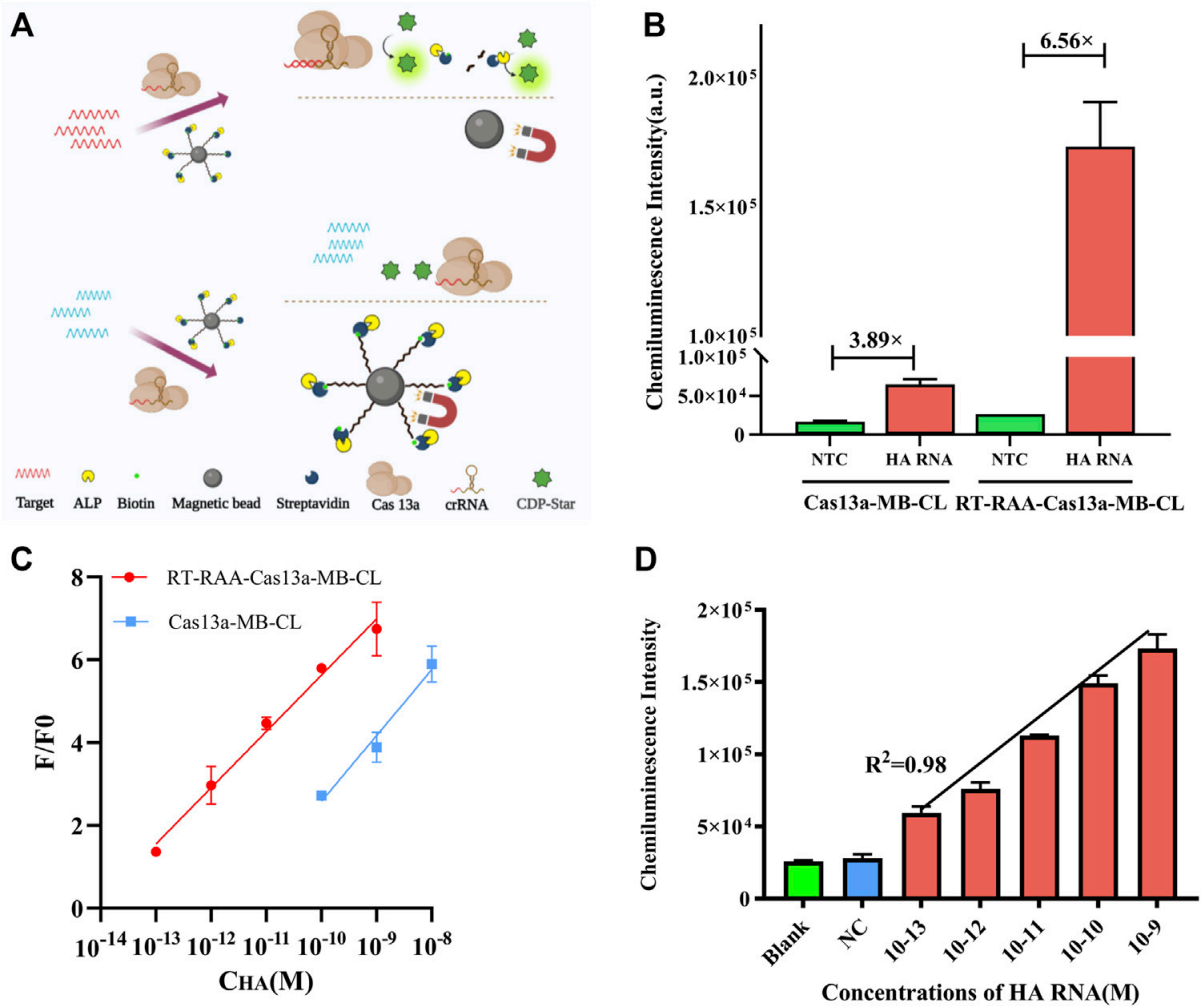
**(A)** Schematic illustration of Cas13a-MB-CL in response to HA RNA. **(B)** CL intensity responses of HA RNA (1 nM) in RT-RAA-Cas13a-MB-CL and Cas13a-MB-CL. **(C)** Calibration curve of RT-RAA-Cas13a-MB-CL (red) and Cas13a-MB-CL (blue) in response to different concentrations of HA RNA. F and F0 are the CL intensities with and without the presence of HA RNA. **(D)** Linear plot of RT-RAA-Cas13a-MB-CL of HA RNA at concentrations from 100 fM to 1 nM. The data are presented as mean ± S.D. of three replicate measurements.

The schematic of Cas13a-MB-CL detection system was illustrated in Figure 3A. The detection limit of Cas13a-MB-CL detecting HA RNA was 100 p.m. (Figure 3C). Besides, Cas13a-MB-CL was used to detect NA RNA, having the same detection limit (Supplementary Figure S1). RT-RAA assay is a newly developed technology which can react at constant temperature and within a short reaction time (30 min) (Wang et al., 2022; Wu et al., 2022). Hence, we combined the CRISPR/Cas system with RT-RAA to improve sensitivity to detect H7N9. The results in Supplementary Figure S2 showed that the RT-RAA amplification system (RT-RAA-Cas13a-MB-CL) did have an effect on the cleaving system, and the CL intensity of positive group was gradually enhanced from the original multiplier products to the 16-fold dilution of products, weakened in 32-fold dilution of products. Therefore, the products of the 16-fold dilution were selected as the optimal optimization condition to be added to the cleaving system (Supplementary Figure S2). The proposed RT-RAA-Cas13a-MB-CL has a much better detection sensitivity compared to Cas13a-MB-CL assay in theory. Then, we investigated CL signals between the two methods in response to different concentrations of HA RNA (Figures 3B,C) and NA RNA (Supplementary Figure S1). In Figure 3B, the CL response signals of 1 nM HA RNA from RT-RAA-Cas13a-MB-CL and Cas13a-MB-CL assay were determined to be 6.56 and 3.89 compared to blank controls, respectively, suggesting an efficient signal gain in RT-RAA-Cas13a-MB-CL. And more importantly, RT-RAA-Cas13a-MB-CL was able to decrease the limit of detection (LOD) by approximately three orders of magnitude compared with the Cas13a-MB-CL assay (Figure 3C). Here, F/F0 was defined as the CL response signal, where F and F0 are the CL intensities of each assay with and without the presence of HA/NA RNA, respectively. Moreover, the LOD of RT-RAA-Cas13a-MB-CL is one to two orders of magnitude lower than that of Cas13a-fluorescence based RNA assays (Abudayyeh et al., 2016). To calculate the LOD of RT-RAA-Cas13a-MB-CL, a linear relationship between the CL response signal and the logarithmic value of H7N9 RNA concentration was obtained in the concentration range from 100 fM to 1 nM, with a LOD of 19.7 fM (3S/N, *R* ^2^ = 0.98) (Choengchan et al., 2006; nanocomposite, 2020) (Figure 3D, Supplementary Figure S3).

### 3.5 Clinical sample testing and specificity verification

**FIGURE 4:**
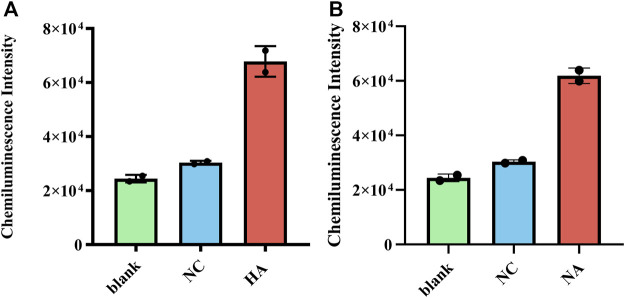
**(A)** Clinical samples using RT-RAA-Cas13a-MB-CL to detect HA RNA and **(B)** NA RNA of H7N9. The data are presented as mean ± S.D. of three replicate measurements; NC, negative control.

The clinical performance of RT-RAA-Cas13a-MB-CL was evaluated using H7N9 virus samples donated by Jiangsu Provincial Center for Disease Prevention and Control. And the CL values of HA and NA in the two clinical samples were greater than that of negative controls (Figures 4A,B), showing a superior sensitivity and specificity.

**FIGURE 5:**
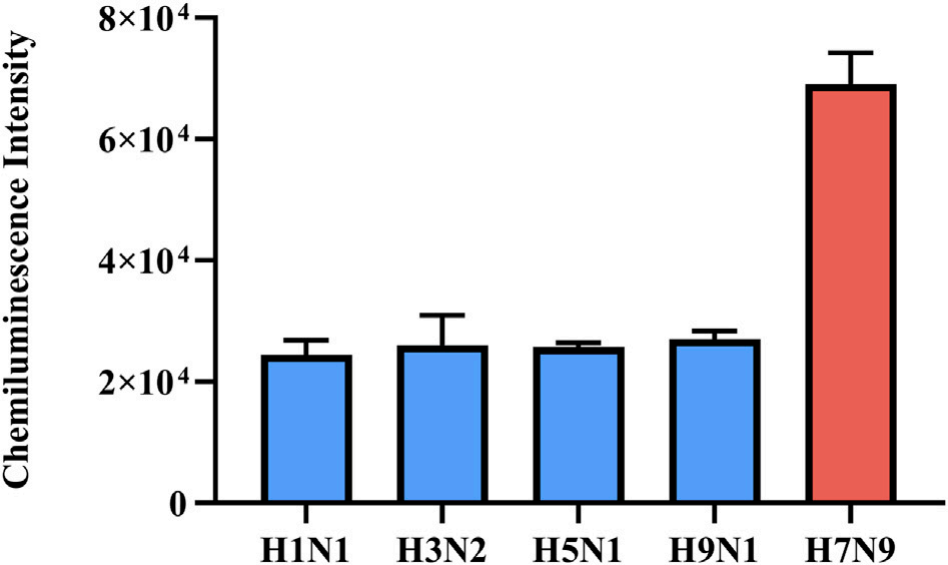
Specificity of RT-RAA-Cas13a-MB-CL detection of H7N9 virus samples against other influenza virus samples for HA. The data are presented as mean ± S.D. of three replicate measurements.

In addition, the detection of H7N9 RNAs by the RT-RAA-Cas13a-MB-CL should be sequence-specific, so we tested seasonal influenza viruses (A/H1N1 and A/H3N2), 2009 swine-origin influenza virus A/H1N1, and avian influenza viruses (A/H5N1 and A/H9N1) donated by Jiangsu Provincial Center for Disease Prevention and Control (Sup. Table 1). The CL detection signals of H7N9 virus samples were significantly higher compared to other virus samples (Figure 5, Supplementary Figure S4). Above all, the proposed method had high specificity in the detection of H7N9 RNAs. Compared with RT-qPCR, the specificity of this method was comparable or even much better. Although slightly less sensitive than RT-qPCR, the entire detection process of this method can be completed in 30 min, while RT-qPCR needs more than 1 hour.

## 4 Conclusions

We successfully developed a novel method for detection of H7N9 virus based on MBs, CL and CRISPR-Cas13a, overcoming the problem of high fluorescence background in CRISPR combined with fluorescence detection technology. We improved sensitivity of detection methods with RT-RAA, a novel developed amplification technique which can react at constant temperature and is compatible with CRISPR. The LOD was as low as 19.7 fM at 25°C. Compared to the traditional fluorescence detection technology, it is an innovation in the method. Since MB-RNA-ALP complexes can be prepared and stored in advance, the entire detection process can be shortened to 30 min. More importantly, this method combined with magnetic bead method has proven to be applicable for influenza virus RNA detection in complex biological samples although having an incompatible reaction system. This assay has great promise for virus detection and can be adapted to clinical large-scale screening in the future. Thus, it is expected to scale up clinical applications and can be a valuable improvement towards achieving automated testing platforms for application in clinical setups in the near future.

## Data Availability

The raw data supporting the conclusions of this article will be made available by the authors, without undue reservation.
