# Supplementary material for: Clustered Regularly Interspaced Short Palindromic Repeats-Associated Proteins13a combined with magnetic beads, chemiluminescence and reverse transcription-recombinase aided amplification for detection of avian influenza a (H7N9) virus
Source: Front Bioeng Biotechnol. 2023 Jan 5;10:1094028. doi: 10.3389/fbioe.2022.1094028 (PMC9849363; doi:10.3389/fbioe.2022.1094028)
Supplement: Supplementary file 1 [file Table1.DOCX]

Supplementary Material

**CRISPR-Cas13a combined with magnetic beads and chemil****uminescence for detection of** **avian influenza A(****H7N9) virus**

Hongpan Xu^1^ †, Lijun Peng^1,2^ †, Jie Wu ^1^ †, Yifan Sun ^1^, Han Shen ^1 *^, Zhiyang Li ^1 *^

^1^ Nanjing Drum Tower Hospital Clinical College of Jiangsu University, Nan Jing, China

^2^ Clinical Laboratory Center, Affiliated Hangzhou Chest Hospital, Zhejiang University School of Medicine, Hangzhou, Zhejiang, P.R. China

*** Correspondence:**Corresponding Author Han Shen *, Zhi yang Li *
[shenhan10366@sina.com](mailto:shenhan10366@sina.com), [lizhiyangcn@qq.com](mailto:lizhiyangcn@qq.com)

†These authors contributed equally to this work and share first authorship

# Supplementary Figures and Tables

## Supplementary Figures


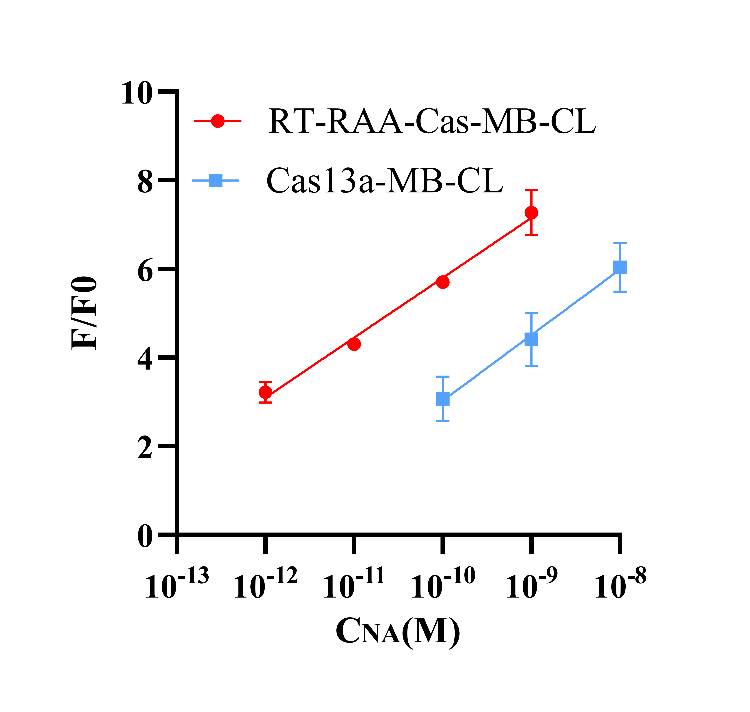


**Supplementary Figure 1** Calibration curve of RT-RAA-Cas13a-MB-CL (red) and Cas13a-MB-CL (blue) in response to different concentrations of NA RNA. The data are presented as mean ±S.D. of three replicate measurements.


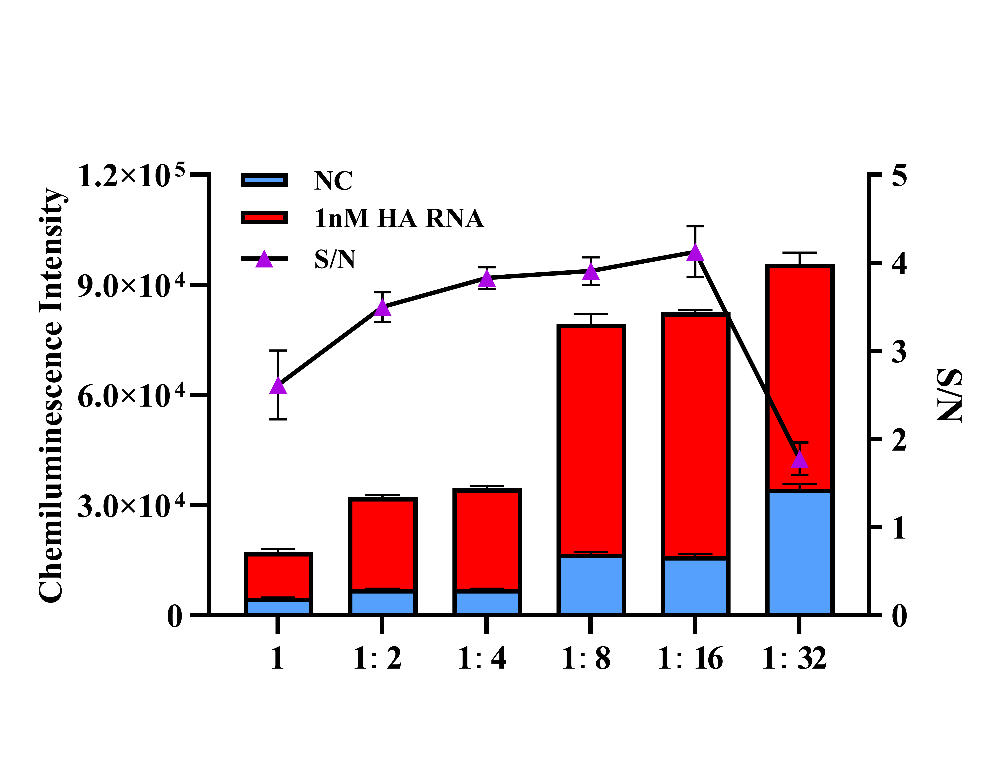


**Supplementary Figure 2 RT-RAA products with different dilution.** The amplified products of RT-RAA were diluted by multiple gradients. The data are presented as mean ±S.D. of three replicate measurements.


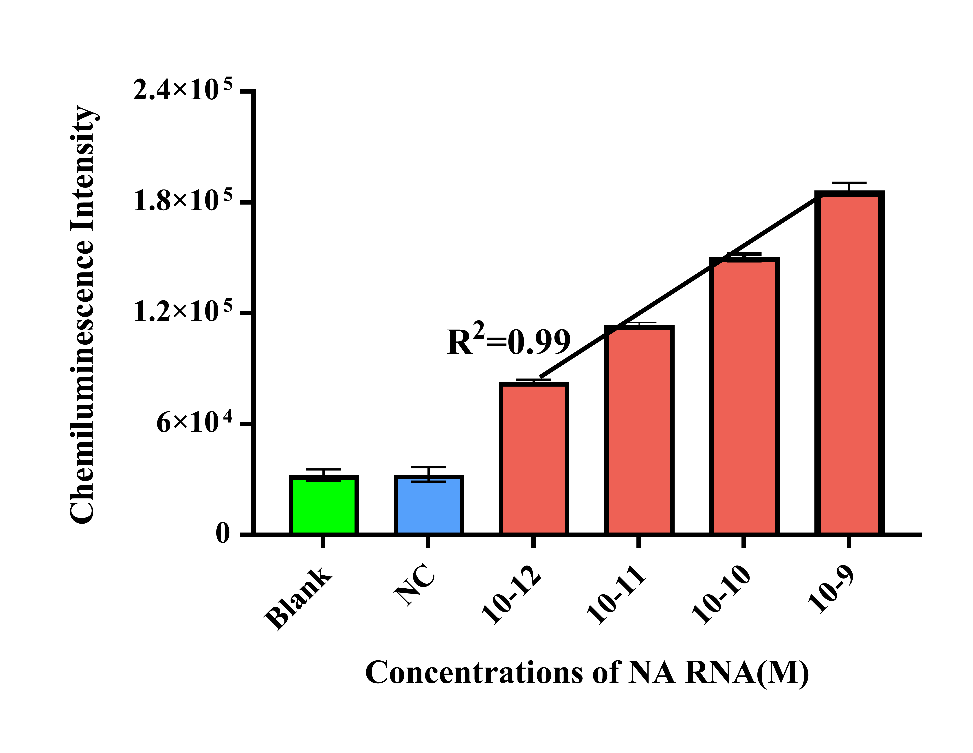


**Supplementary Figure 3** Linear plot of RT-RAA-Cas13a-MB-CL of NA RNA at concentrations from 1pM to 1 nM. The data are presented as mean ±S.D. of three replicate measurements.


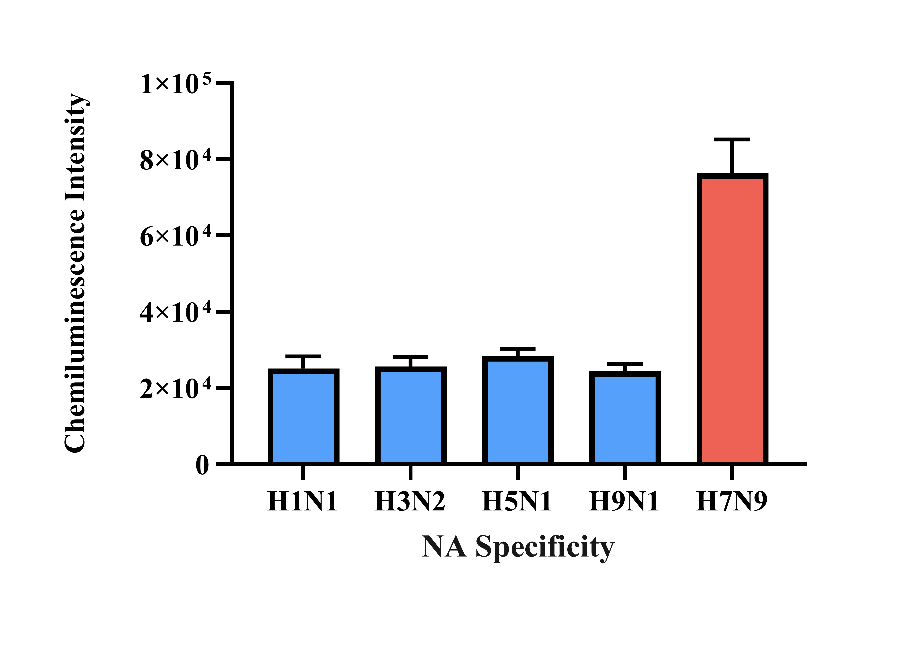


**Supplementary Figure 4** Specificity of RT-RAA-Cas13a-MB-CL detection of H7N9 virus samples against other influenza virus samples for NA. The data are presented as mean ±S.D. of three replicate measurements

**Supplementary Table**

| **Supplementary Table.1** | |
| --- | --- |
| Name | Sequence |
| HA-F | CCTGGTATTCGCTCTGATTGC （21nt） |
| HA-R | TAATACGACTCACTATAGGGAGGCACCGCATGTTTCCATTCT （42nt） |
| HA-crRNA-DNAF | TAATACGACTCACTATAGGGGATTTAGACTACCCCAAAAACGAAGGGGACTAAAACGATTGACCCAGTCAAACTAAGCAGCGGC （84nt） |
| HA-crRNA-DNAR | TAATACGACTCACTATAGGGGATTTAGACTACCCCAAAAACGAAGGGGACTAAAACGATTGACCCAGTCAAACTAAGCAGCGGC（84nt） |
| HA-crRNA | rGrGrGrGrArUrUrUrArGrArCrUrArCrCrCrCrArArArArArCrGrArArGrGrGrGrArCrUrArArArArCrGrArUrUrGrArCrCrCrArGrUrCrArArArCrUrArArGrCrArGrCrGrGrC（67nt） |
| NA-F | GAAACAACCAACACAAGCCA（20nt） |
| NA-R | TAATACGACTCACTATAGGGCATCCGAGCTTTCTCCAATTCTT（43nt） |
| NA-crRNA-DNAF | TAATACGACTCACTATAGGGGATTTAGACTACCCCAAAAACGAAGGGGACTAAAACTCCAAATAGAAGAAAGAACAAGCAGGAA（84nt） |
| NA-crRNA-DNAR | TTCCTGCTTGTTCTTTCTTCTATTTGGAGTTTTAGTCCCCTTCGTTTTTGGGGTAGTCTAAATCCCCTATAGTGAGTCGTATTA（84nt） |
| NA-crRNA | rGrGrGrGrArUrUrUrArGrArCrUrArCrCrCrCrArArArArArCrGrArArGrGrGrGrArCrUrArArArArCrUrCrCrArArArUrArGrArArGrArArArGrArArCrArArGrCrArGrGrArA（67nt） |
| RNA probes | /5‘AminoC6/rUrCrGrGrUrUrUrGrUrArArGrCrCrArGrUrUrUrUrUrCrGrUrCrArArUrCrArGrUrUrUrUrUrCrUrArArUrCrGrGrCrCrArGrU/3’Biotin/（49nt） |

*F: Forward primer; R: Reverse primer.
